# Neurofilament accumulation disrupts autophagy in giant axonal neuropathy

**DOI:** 10.1172/jci.insight.177999

**Published:** 2025-03-10

**Authors:** Jean-Michel Paumier, James Zewe, Chiranjit Panja, Melissa R. Pergande, Meghana Venkatesan, Eitan Israeli, Shikha Prasad, Natasha Snider, Jeffrey N. Savas, Puneet Opal

**Affiliations:** 1 Davee Department of Neurology, Feinberg School of Medicine, Northwestern University, Chicago, Illinois, USA.; 2 Department of Cell Biology and Physiology, School of Medicine, University of North Carolina at Chapel Hill, North Carolina, USA.; 3 Department of Cell and Molecular Biology, Northwestern University, Chicago, Illinois, USA.

**Keywords:** Cell biology, Neuroscience, Autophagy, Neurological disorders, Ubiquitin-proteosome system

## Abstract

Neurofilament accumulation is associated with many neurodegenerative diseases, but it is the primary pathology in giant axonal neuropathy (GAN). This childhood-onset autosomal recessive disease is caused by loss-of-function mutations in gigaxonin, the E3 adaptor protein that enables neurofilament degradation. Using a combination of genetic and RNA interference approaches, we found that dorsal root ganglia from mice lacking gigaxonin have impaired autophagy and lysosomal degradation through 2 mechanisms. First, neurofilament accumulations interfere with the distribution of autophagic organelles, impairing their maturation and fusion with lysosomes. Second, the accumulations attract the chaperone 14-3-3, which is responsible for the proper localization of the key autophagy regulator transcription factor EB (TFEB). We propose that this dual disruption of autophagy contributes to the pathogenesis of other neurodegenerative diseases involving neurofilament accumulations.

## Abstract 2

Giant Axonal Neuropathy is caused by mutations in gigaxonin, a protein that degrades neurofilaments. Neurofilament accumulation impairs autophagy by tethering autophagic vesicles and TFEB.

## Introduction

Giant axonal neuropathy (GAN) affects virtually every facet of the peripheral and central nervous systems (1, 2). Patients initially present with weakness, wasting, and sensory deficits in a glove-and-stocking distribution. As the disease progresses, patients suffer cognitive decline, cranial nerve dysfunction, seizures, spasticity, and cerebellar incoordination; most patients do not live beyond the third decade of life. The distal-to-proximal pattern of deterioration reflects the greater susceptibility of neurons with very long axons to the consequences of mislocalization and dysfunction of cytoskeletal proteins known as intermediate filaments (IFs). Interestingly, the accumulation of these proteins, so named because they are larger than microtubules but smaller than actin (3), can be used as a biomarker for the progression of a variety of neurological diseases, from peripheral neuropathies to multiple sclerosis, amyotrophic lateral sclerosis, dementias, and Parkinson and Alzheimer diseases (4, 5). Only in GAN, however, is IF accumulation the primary pathology, and as such this disease can tell us much about IF function and the pathogenic pathways initiated by IF accumulation.

In GAN, the accumulation of IFs is a direct result of biallelic loss-of-function mutations of the *GAN* gene (1, 6), which encodes gigaxonin, a member of the bric-a-brac, tramtrack, and broad (BTB)-kelch family of E3 ligase adaptors that recruit substrates for ubiquitin-mediated proteasomal degradation (7). The N-terminal BTB domain binds cullin 3, which serves as a bridge to the rest of the ubiquitination machinery (8); the C-terminal kelch domain binds select substrates, of which the best characterized are IFs (9–11). IFs are classified into 6 major types (I–VI) based on their primary structure and tissue of expression (3, 12), but all IFs share a tripartite structure that includes variable globular N- and C-terminal domains and a central conserved rod domain. It is this central rod domain to which gigaxonin binds.

Neurons have the most complex repertoire of IFs of any cell type, and they bear the brunt of GAN pathology. All neurons express 3 of the type IV IFs, known as neurofilaments (NFs): neurofilament triplet proteins neurofilament heavy (NFH), middle (NFM), and light (NFL), so named based on their molecular weights. Some neurons also express α-internexin (a type IV IF), while others, particularly those in the peripheral nervous system, express peripherin, a type III IF (13). In GAN, NFs accumulate throughout the cytoplasmic space, but also in discrete foci that are remarkably stable (9, 14). Under magnification, these foci look like a tangle of fibers; for reasons that are not entirely clear, the normal transport of even individual filaments along microtubules in GAN is impaired (15).

Although it seems intuitively obvious that NF accumulations should interfere with the mechanical properties of the neuron and the signaling events that the NFs regulate (13, 16), we have been interested in how NF accumulation affects specific cellular functions and whether other dysregulated proteins are involved in GAN pathogenesis. For example, we previously found that NF accumulations interfere with mitochondrial trafficking, thereby impairing mitochondrial function (9). Here, we investigate the effect of NF accumulation on autophagic processes, which are spatially orchestrated in neurons; in healthy conditions, substrates are engulfed by autophagic vesicles more distally, and then are transported retrogradely from the neurites to the soma, where they are delivered to the lysosomes for degradation (17). We find that NF accumulations alter the spatial distribution of lysosomes, compromising their autophagic activities. We also show that the NF accumulations attract 14-3-3 proteins, a family of chaperone proteins known to bind IFs, along with the master transcriptional regulator transcription factor EB (TFEB), thereby preventing TFEB from shuttling to the nucleus to perform its transcriptional functions. The result is a progressive loss of quality control of both proteins and organelles, causing cellular deterioration.

## Results

### Proteomic analysis of mouse dorsal root ganglia with gigaxonin silenced.

**Figure 1:**
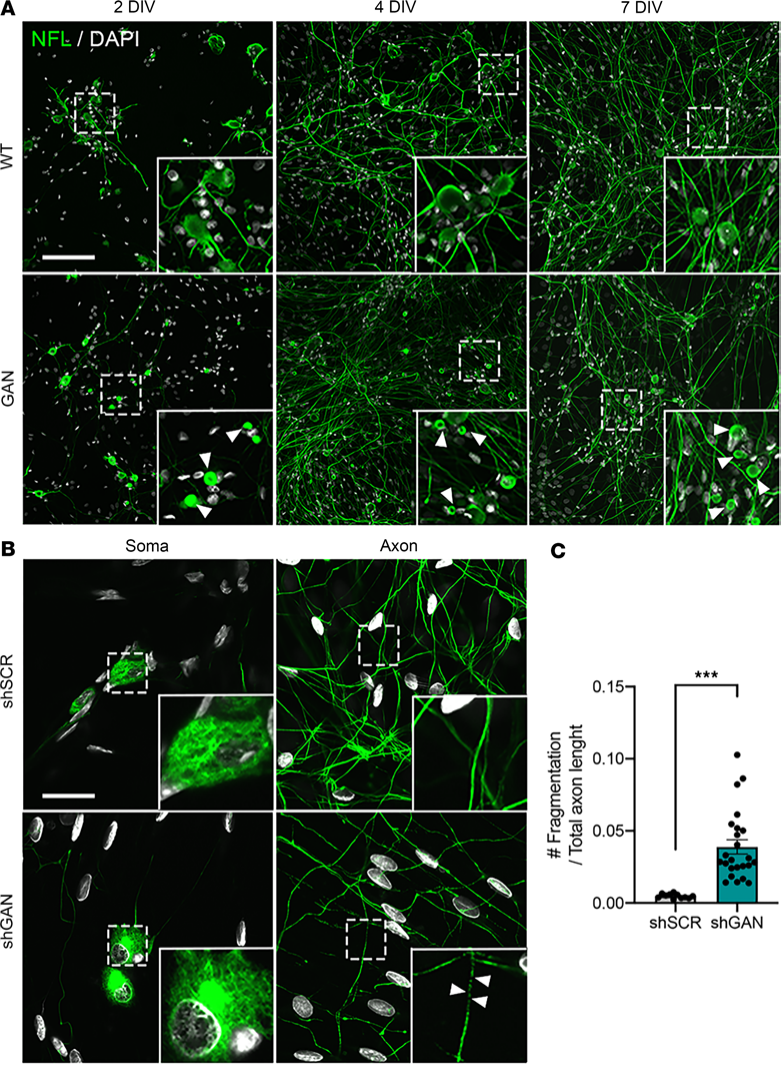
Dorsal root ganglia (DRG) neurons model the hallmark pathology of giant axonal neuropathy (GAN). (**A**) Representative fluorescence microscopy images of DRG neurons from WT and *Gan*-null mice stained for neurofilament light (NFL) after 2, 4, and 7 days in vitro (DIV). Arrowheads denote NFL aggregation in the soma of *Gan*-null DRG neurons. Note that NFL aggregates are already present after 2 DIV. (**B**) The GAN phenotype can be recapitulated by lentiviral delivery of shRNA targeting the *Gan* gene. Scale bars: 30 μm (**A** and **B**). All insets are shown at ×3 magnification. After 12 DIV, axonal fragmentation, a sign of neurodegeneration, occurs in *Gan*-silenced DRG neurons, as denoted by arrowheads; large aggregate shown in zoom. (**C**) Quantification of axonal fragmentation was accomplished using Fiji’s measurement tool, reported here as the means of 3 independent experiments ± SEM. ***P < 0.001 by 2-tailed, unpaired Student’s *t* test.

*Gan*-null mice recapitulate the neurofilament aggregates in neurons and the IF aggregates in other cell types, but because of their small size, they do not manifest overt signs of the disease until they are very old (9, 18–20). Therefore, we developed a primary neuronal culture model using dorsal root ganglia (DRG) neurons (9), which are affected early in the disease course and display severe neuropathology. DRG neurons isolated from *Gan*-null mice demonstrated progressive NF accumulation starting from as early as 2 days in vitro, both in the cell soma and neurites (Figure 1A). The GAN phenotype is equally well reproduced in wild-type (WT) neurons using lentiviral delivery of small hairpin RNA–based (shRNA-based) RNAi to reduce gigaxonin expression. The degree of knockdown achieved is approximately 90% (9). Neurons lacking gigaxonin degenerate, as evidenced by axonal fragmentation after an additional 8–9 days in culture (Figure 1, B and C).

**Figure 2:**
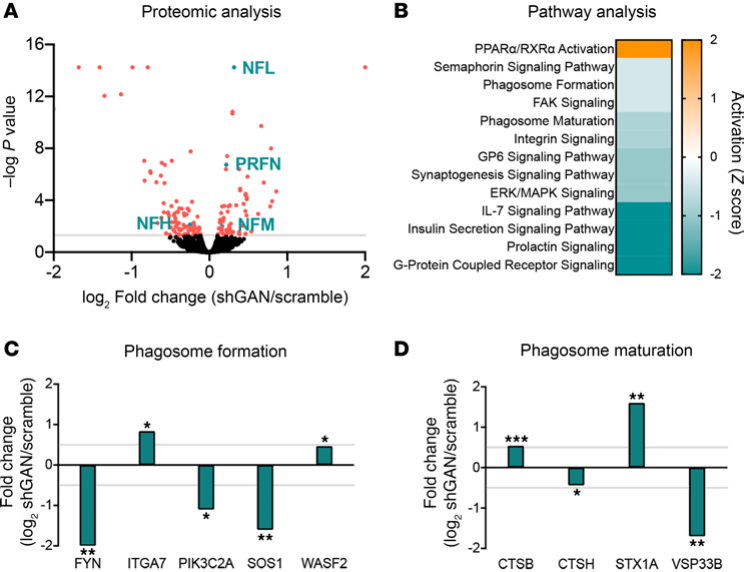
Mass spectrometry–based proteomic analysis of dorsal root ganglia (DRG) cultures silenced for gigaxonin. (**A**) Volcano plot showing the distribution of measured proteins extracted from DRG cultures in which *Gan* was silenced using shRNA (shGan). (**B**) Plot showing top-ranked altered pathways in GAN where pathway activation (change in *z* score) is shown (orange represents upregulation and blue represents downregulation). (**C** and **D**) Plots showing altered proteins for the phagosome formation and phagosome maturation pathways. *P < 0.05, **P < 0.001, ***P < 0.0001; adjusted *P* values from 2-tailed, unpaired Student’s *t* test. NFL, neurofilament light; NFM, neurofilament medium; PRFN, peripherin.

To gain insight into the molecular consequences of loss of gigaxonin function, we performed quantitative proteomics with stable isotope labeling using amino acids in cell culture (SILAC) (21). We quantified 3,507 proteins in DRG cultures (shScr) and *Gan*-knockdown cells (shGan) under the same experimental conditions (Figure 2A). Of these, 149 proteins had significantly altered fold change, with a false discovery rate–adjusted (FDR-adjusted) *P* value of less than 0.05 (62 elevated and 87 reduced; see Supplemental Table 1; supplemental material available online with this article; https://doi.org/10.1172/jci.insight.177999DS1). As expected, the IF proteins (NFL, NFM, and peripherin) were among those that were most significantly elevated.

Ingenuity Pathway Analysis (IPA) identified altered biological functions in *Gan*-silenced DRG cultures (Figure 2B). Several signaling pathways were dysregulated, but only 1 was hyperactivated: PPARα/RXRα, which regulates cell growth, differentiation, and metabolism. Since signaling pathways participate in multiple cellular functions, it was difficult to discern which specific deficits might result. We noticed, however, that phagosome formation and maturation, 2 key aspects of autophagy (22), were downregulated. Among the downregulated proteins involved in these functions were Fyn, which plays a role in autophagy by AMPK phosphorylation; PIK3C2A, whose knockdown decreases autophagy and the maturation of endocytic vesicles (23); and Sos1, whose deletion has been related to accumulation of phagosomes and lysosomal bodies (24) (Figure 2C). There were also genes in these autophagy pathways that were upregulated, perhaps in compensation, such as Itga7, which participates in phagocytosis (25), and Wasf2, which is involved in autophagosome and trafficking to lysosomes in human immune cells (26). Two upregulated proteins in the phagosome maturation category (Figure 2D) were cathepsin B (Ctsb), whose deletion impairs autophagy and lysosomal recycling (27), and syntaxin 1A (Stx1A), which regulates vesicle fusion and trafficking (28); among the downregulated proteins were cathepsin H (Ctsh), which is also involved in vesicle fusion and trafficking (29), and VPS33B, which is involved in endosomal recycling and late endosomal-lysosomal fusion events (30, 31). Several of the other dysregulated pathways, most notably PPARα/RXRα, ERK/MAPK, IL-7, semaphorin, and GPCR signaling, are also central to autophagic regulation (32–35). The overall impression is thus of a severely dysregulated autophagic system. Our proteomic results are also supported by a recent publication describing the role of gigaxonin in autophagosome production through Atg16L1 turnover regulation (36).

### NF aggregates alter the spatial distribution, abundance, and morphology of autophagic organelles.

**Figure 3:**
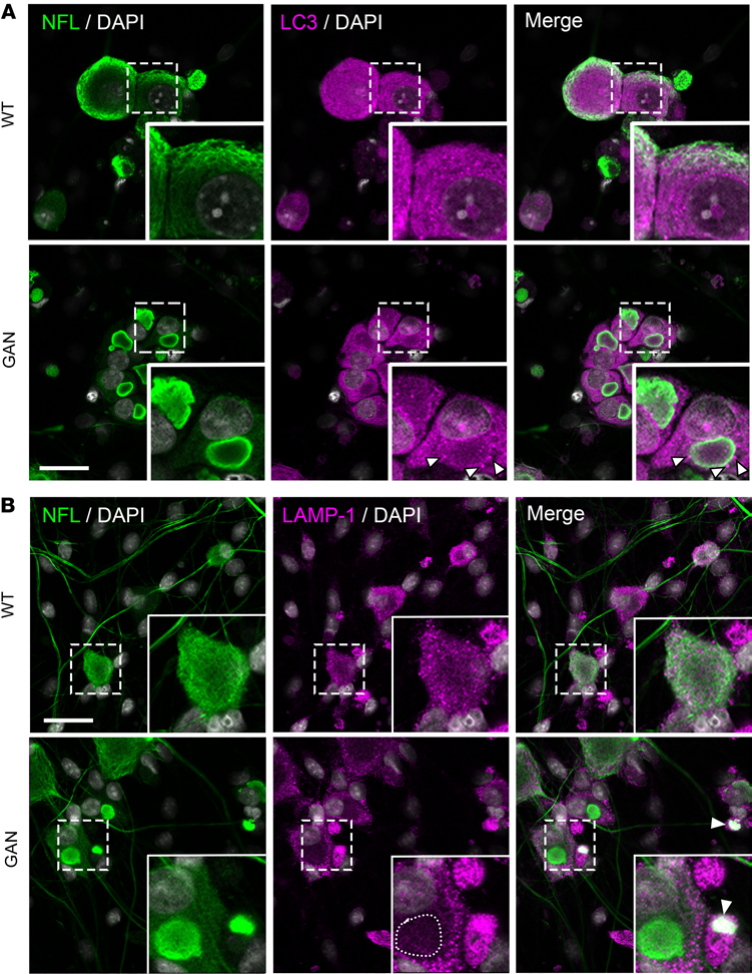
Neurofilament aggregates alter autophagosome spatial distribution. (**A**) Representative fluorescence images of DRG neurons from WT or *Gan*-null mice costained for the cytoskeleton marker neurofilament light (NFL) and the autophagosome marker LC3. Autophagosomes are excluded from neurofilament aggregates, and LC3 puncta are found at the periphery of the aggregates (white arrowhead). (**B**) WT or *Gan*-null mouse DRG neurons costained for the cytoskeletal marker NFL and the lysosomal marker LAMP-1. Two phenotypes are observed in *Gan*-null DRG neurons with neurofilament aggregates: lysosomes are either excluded (circular dotted line) or colocalized with neurofilament aggregates (arrowhead). Scale bars: 30 μm. Insets are shown at ×3 magnification. Representative images from 3 independent experiments.

The autophagic process involves the formation of vesicles around substrates; these vesicles mature into autophagosomes that fuse with lysosomes, where the substrate is ultimately degraded (37). These steps require the free movement of autophagic organelles, a process that we hypothesized would be particularly compromised in neurons by the space-occupying NF aggregates. Therefore, we stained cells for LC3, a small polypeptide that recruits substrates and is a specific marker for autophagosomes (38). NFs were delineated by staining for NFL, a protein that forms the backbone of the NF heteropolymer (16). In *Gan*-null DRG neurons, the autophagosomes were not uniformly distributed throughout the cytoplasm, as they would normally be, but were now more abundant at the perimeters of the aggregates (Figure 3A).

Next, we determined the location of lysosomes using immunofluorescence microscopy. NFs were visualized as before by staining for NFL, while lysosomes were visualized by staining for LAMP-1, which constitutes approximately 50% of the lysosomal membrane (16, 39) (Figure 3B). Neurons showed either exclusion of LAMP-1 staining from NF aggregates, or they showed a colocalization. We surmised that these 2 patterns reflect the status of different lysosomal populations or their membranous fragments. Indeed, there is a growing body of literature on lysosome heterogeneity, both in different cell types and within the same cell (40–42). This heterogeneity involves gradations in the acidity of the interiors of these lysosomes and their repertoire of luminal cathepsins and proteases (27, 40).

**Figure 4:**
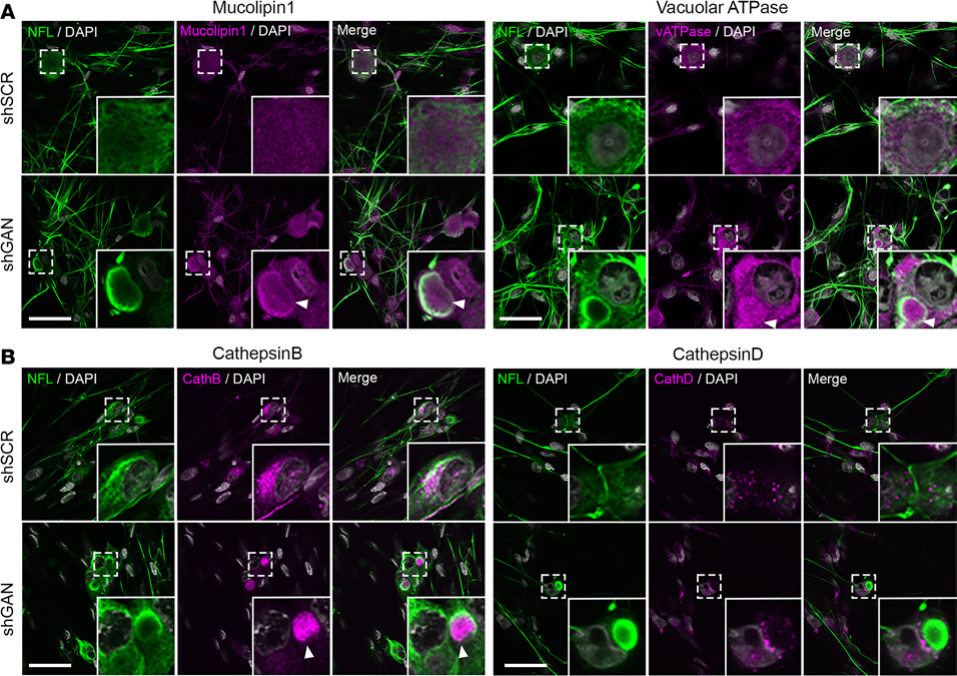
Spatial distribution of lysosomes is altered by neurofilament aggregates in GAN DRG neurons. Representative fluorescence images of DRG neurons silenced for gigaxonin (shGan) and control neurons (shScr) costained for NFL and (**A**) mucolipin-1 and vacuolar ATPase or (**B**) cathepsin B and cathepsin D. While cathepsin D is excluded from neurofilament aggregates, the 3 other lysosomal proteins colocalize with aggregates in shGan cells from mouse DRG. Scale bars: 30 μm. Insets are shown at ×3 magnification. Arrowheads highlight lysosomal proteins clumped in NFL aggregates in the shGan condition.

To follow up on these observations, we stained additional lysosomal components, both across the membrane and within the lumen. For the former, we evaluated the distribution of mucolipin-1, a calcium channel protein and member of the transient receptor potential cation channel mucolipin subfamily (43, 44), and vacuolar ATPase, which is a protein essential for lysosomal acidification that pumps protons into the lumen (45, 46). Both of these colocalized with NF aggregates (Figure 4A). To study intraluminal proteins, we stained for 2 lysosomal proteases: cathepsin B and cathepsin D (47). Cathepsin B was typically found in aggregates, whereas cathepsin D was typically excluded from them, reminiscent of the 2 staining patterns of LAMP-1 and consistent with lysosomal heterogeneity (Figure 4B). It is also worth noting that cathepsin D, which requires a more acidic pH for its activity (pH 4.5–5), is seen in the discrete intact lysosomes, while cathepsin B, which is active at a less acidic pH (pH 5–6), appears to be more closely packed within the NF aggregates (27).

**Figure 5:**
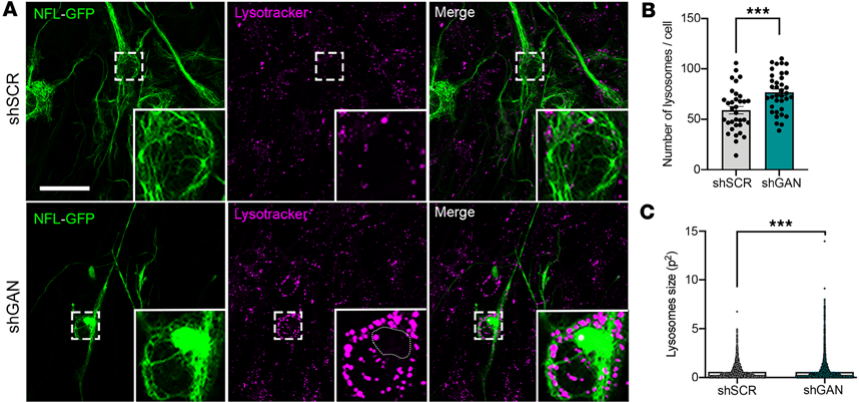
Lysosomal changes in GAN. (**A**) Representative live-imaging fluorescence images of control and shGan cells from mouse DRG transduced with NFL-GFP tagged lentivirus and treated with red LysoTracker to visualize lysosomes. Expression of the NFL-GFP construct in shGan cells from mouse DRG neurons allowed neurofilament aggregate visualization in living cells. Scale bar: 30 μm. Insets are shown at ×3 magnification. (**B** and **C**) shGan induces an increase in the number of lysosomes and the surface area covered by these organelles. Note that as observed after NFL and LAMP-1 costaining, LysoTracker dye is also mainly excluded from neurofilament aggregates (circular dotted line). Quantitative data are presented as mean ± SEM. ***P < 0.001 by 2-tailed, unpaired Student’s *t* test. Representative images from 3 independent experiments.

We next tracked intact lysosomes in living DRG neurons with LysoTracker, a cell-permeable dye that stains the acidic compartment of lysosomes across a range of pH levels (48). Since we wished to correlate lysosomal distribution with NF aggregates, we also infected cells with a lentivirus expressing GFP-tagged NFL. LysoTracker staining tended to be absent from regions with aggregates, suggesting that intact lysosomes are spatially excluded from NF accumulations (Figure 5A). The amount and intensity of lysosomal staining with LysoTracker was greater in shGan cells compared with controls (Figure 5, A and B), likely because the lysosomes also tended to be larger (Figure 5C).

**Figure 6:**
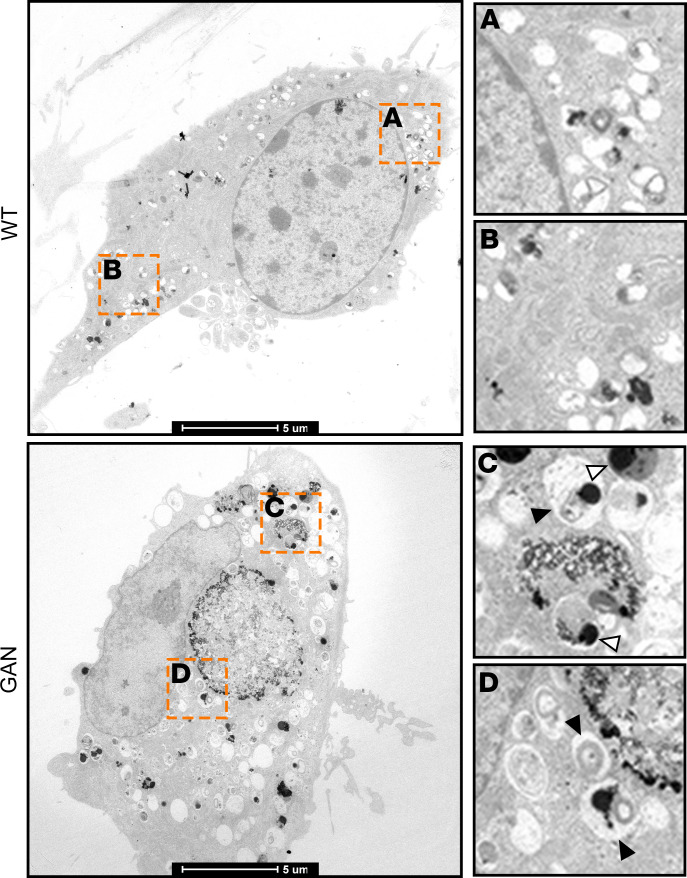
GAN is associated with abnormal autophagic organelles. Transmission electronic microscopy microphotographs of control and *Gan*-null DRG neurons showing the presence of a variety of autophagic organelles, including large autophagic vacuoles, dark dense lysosomes (open arrowheads), multilamellar bodies, and immature autophagosomes (filled arrowheads). Scale bars: 5 μm. Insets are shown at ×3.5 magnification.

Transmission electron microscopy revealed a greater abundance of autophagic organelles at different stages of maturation in *Gan*-null DRG neurons; these included large autophagic vacuoles, multilamellar bodies, and immature autophagosomes (Figure 6). The electron-dense lysosomes tended to decorate the perimeter of the aggregates. These data demonstrate that NF aggregates influence the distribution of autophagic organelles.

### Lysosomal acidity and autophagic flux are dysregulated in GAN.

**Figure 7:**
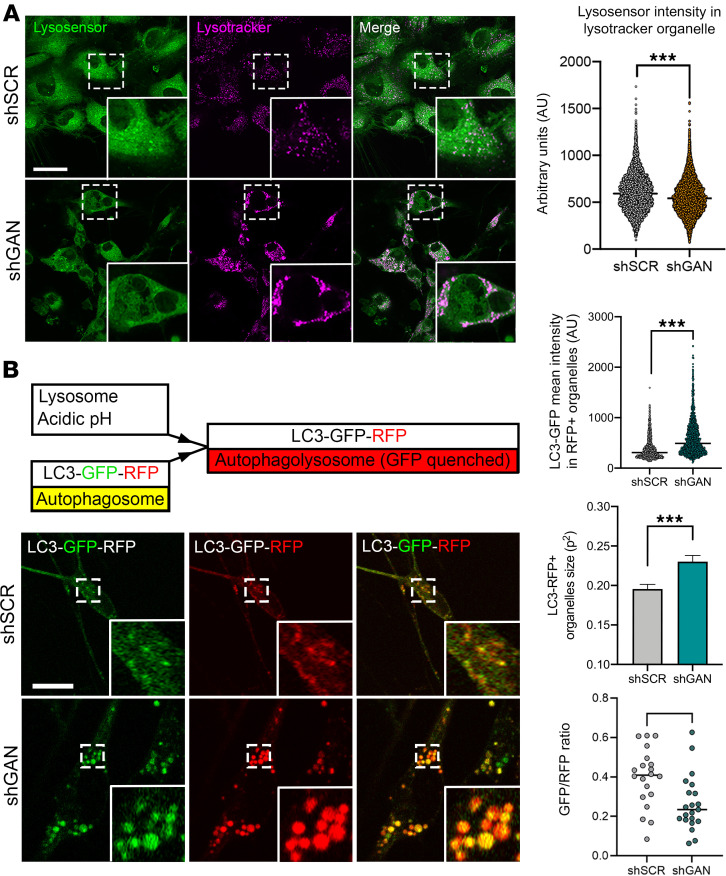
Lysosomal acidity and function are dysregulated in GAN. (**A**) Representative live-imaging microphotographs of control and shGan silenced DRG cells treated with a combination of red LysoTracker to visualize lysosomes and green LysoSensor to evaluate pH changes. Mean intensity of LysoSensor (pH-sensitive probe) is decreased in lysosomes from shGan cultures, suggesting the lysosomal milieu is less acidic in the GAN condition. (**B**) Representative live-imaging microphotographs of control or shGan DRG cells transduced with the sensor LC3-GFP-RFP. Individual panels are presented for GFP, RFP, and merged signals. The construct shown above is composed of an RFP pH-resistant tag, a GFP pH-sensitive tag, and LC3 that targets the tags to nascent autophagosomes. After fusion with lysosomes, autophagolysosomes are formed and the GFP signal is quenched due to the acidic milieu provided by lysosomes, converting the fluorescence signal from yellow to red. Gigaxonin reduction is associated with enhanced GFP fluorescence signal within RFP-positive vesicles; an increase in the size of autophagic organelles; and a decrease in the GFP/RFP ratio. Quantitative data are presented as mean ± SEM. ***P < 0.001 by 2-tailed, unpaired Student’s *t* test. Scale bars: 30 μm. Insets are shown at ×3 magnification.

To address lysosomal function, we evaluated lysosomal pH, which is crucial to the ability to degrade substrates. We used LysoSensor, a sensitive dye designed specifically for this purpose (37). *Gan*-silenced DRG cultures displayed a significant reduction in LysoSensor signal intensity within LysoTracker-defined compartments (Figure 7A). These results suggest that lysosomes in GAN are defective at maintaining a robust acidic internal environment, which would translate into a reduction in autophagic flux. To test this possibility, we performed live-cell imaging using an mRFP-GFP tandem–tagged LC3. This protein is incorporated into the membrane of autophagic vacuoles, exhibiting a punctate signal within cells. It fluoresces from fluorophores as autophagosomes mature before fusing with the lysosome; after fusion, the GFP fluorescence (which is pH sensitive) is lost in the acidic lysosomal milieu, while the mRFP fluorescence (not pH sensitive) persists until LC3 is fully degraded, providing a quantifiable readout for fusion delay or lysosomal dysfunction (38). Neurons lacking gigaxonin showed a significantly greater GFP fluorescence signal within RFP-positive vesicles (Figure 7B). These autophagic organelles were larger in *Gan*-null DRG neurons than in controls. Moreover, we observed a decrease in the GFP/RFP ratio, suggesting that not only is the GFP not quenched, but the RFP-LC3 is also not degraded.

**Figure 8:**
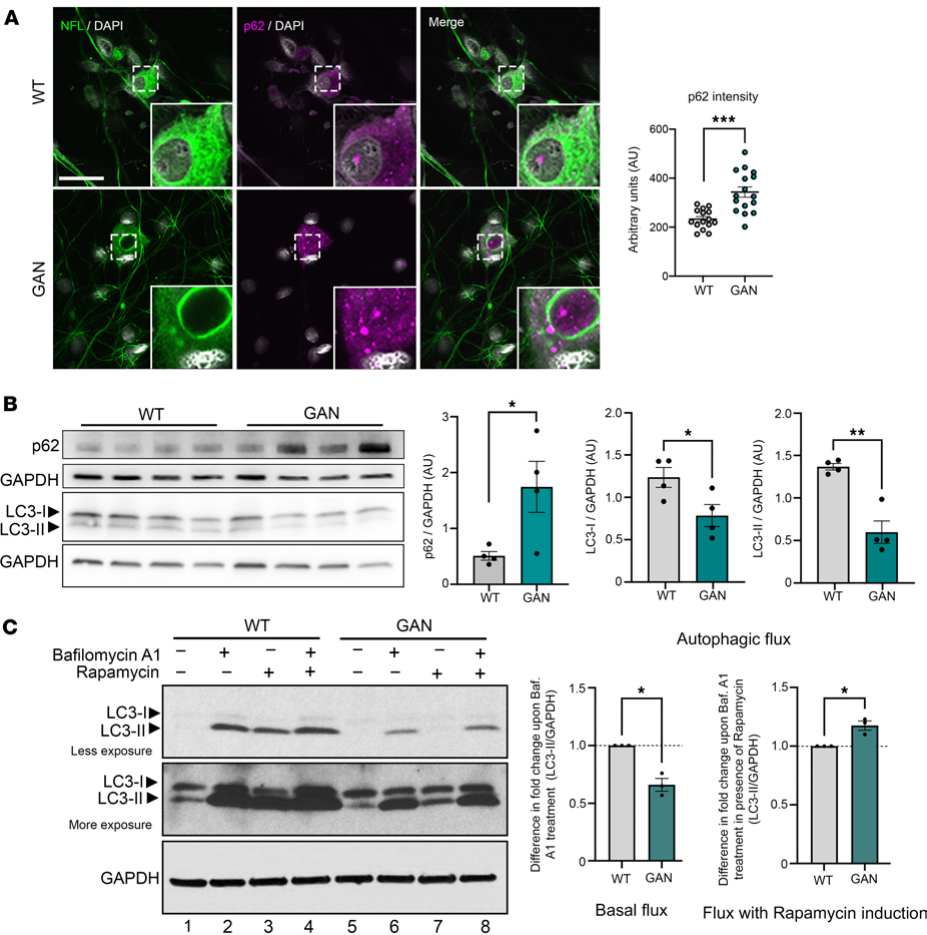
Autophagic flux is dysregulated in GAN. (**A**) The autophagic receptor p62 is increased in *Gan*-null DRG cultures. Representative fluorescence images of control and *Gan*-null DRG neurons costained for p62 and NFL, with mean intensity quantified. Scale bar: 30 μm. Insets are shown at ×3 magnification. (**B**) Western blots show an increase in p62 and a reduction in LC3 isoforms LC3-I and LC3-II; quantified in adjacent histograms. (**C**) Autophagic flux is downregulated in *Gan*-null DRG cultures. DRG neurons from WT and *Gan*-null mice were treated with vehicle, bafilomycin A1, rapamycin, and both drugs as shown. Basal autophagic flux was calculated as the difference between the levels of LC3-II expression (normalized to GAPDH as a loading control) in the bafilomycin A1 treatment in each experimental condition compared to its levels treated with vehicle control. Autophagic flux under rapamycin stimulation is shown alongside. Autophagic flux induced by rapamycin was calculated as the difference between the levels of normalized LC-II expression in the combination rapamycin and bafilomycin A1 treatment and those treated with rapamycin alone. *n* = 3, with sample Western blot of 1 representative experiment shown. The LC3 blot is shown at short and long exposure to clearly display bands. Quantitative data are presented as mean ± SEM. *P < 0.05, **P < 0.01, ***P < 0.001 by 2-tailed, unpaired Student’s *t* test.

To further assess the functional status of autophagy, we evaluated the levels of p62 (also known as SQSTM1), an autophagic receptor that recruits cargo to be degraded and is itself degraded by autophagy (38). By both immunostaining and Western blotting, p62 levels were significantly greater in *Gan*-silenced DRG neurons (Figure 8, A and B). We also observed a reduction in both isoforms of LC3 in *Gan*-null DRG neurons involved in autophagy; (Figure 8B; LC-I is the inactive form, while LC3-II is an activated lipidated form of LC-1, which is recruited to the membranes of autophagosomes to promote autophagy) (38).

Since autophagic processes are dynamic and cannot be monitored simply by the steady-state levels of autophagic markers (49–52), we performed additional biochemical experiments. For these experiments we used bafilomycin A1, a drug that prevents the fusion of autophagosomes with lysosomes and leads to the accumulation of autophagosomes. This leads to an increase in LC-II levels, which indicates the degree of basal autophagic flux (as it shows the amount of LC-II that would normally be degraded by the lysosomes). *Gan*-null DRG neurons showed lower basal autophagic flux than WT neurons (Figure 8C). We also assayed for autophagic flux in the presence of rapamycin, which inhibits mTOR and induces autophagy by promoting autophagosome formation. Under these conditions, *Gan*-null DRG neurons showed a considerable increase in autophagic flux compared even with WT neurons, suggesting that autophagic stimulation could well prove neuroprotective (Figure 8C).

### TFEB localizes to NF aggregates in GAN.

Autophagy is dependent on the activity of TFEB (53–55), a key transcriptional regulator of autophagy, whose activity is highly dependent on its subcellular location. Under conditions of cellular stress, TFEB translocates to the nucleus to drive the coordinated lysosomal expression and regulation (CLEAR) network of genes responsible for autophagy and lysosomal biogenesis (56). When phosphorylated, TFEB is bound to the cytoplasmic chaperone 14-3-3 proteins, a family of acidic phosphoproteins (28–33 kDa in size) that serve as adapters regulating a number of signaling pathways. Intriguingly, IFs, including NFs, are known to recruit these proteins in a phosphorylation-dependent manner (57). For these reasons, we decided to look for both TFEB and 14-3-3 localization in *Gan*-silenced DRG neurons.

**Figure 9:**
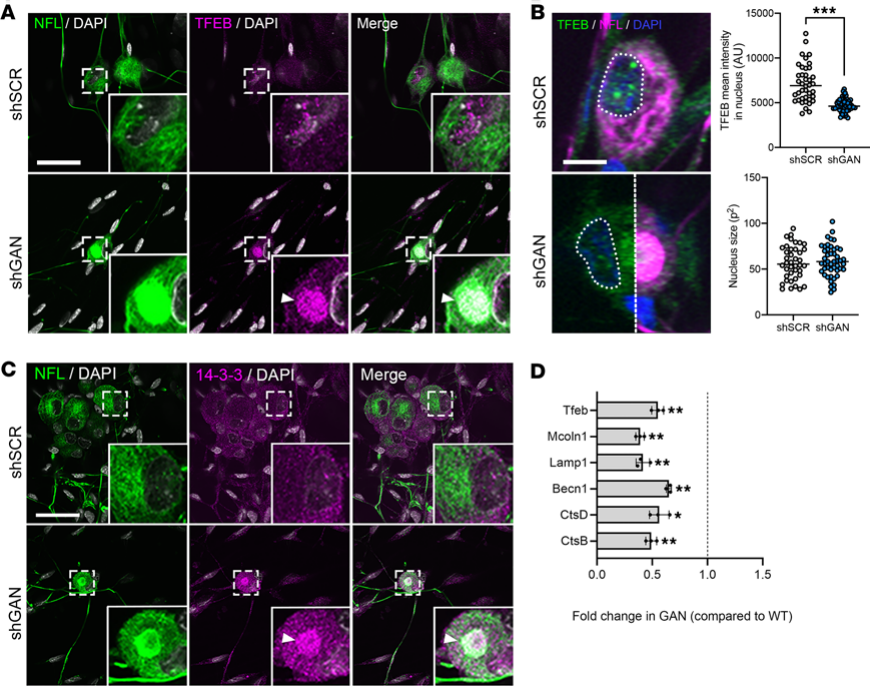
Neurofilament aggregates recruit TFEB and impair its nuclear translocation. (**A**) Representative fluorescence images of control and shGan cells from DRG neurons costained for NFL and TFEB showing colocalization of transcription factor EB (TFEB) to neurofilaments (arrowhead). Scale bar: 30 μm. (**B**) Higher-magnification pictures showing decreased TFEB localization in the nuclear compartment in shGan cells. Accompanying plots show that TFEB has decreased intensity, and that nuclear size is similar between control and shGan cells. Quantitative data are presented as mean ± SEM. ***P < 0.001 by 2-tailed, unpaired Student’s *t* test. (**C**) Immunofluorescence images of control and shGan cells from mouse DRG costained for NFL and 14-3-3. Neurofilament aggregates recruit 14-3-3 (arrowhead). Scale bars: 30 μm. Insets are shown at ×3 magnification. Representative images from 3 independent experiments. (**D**) qRT-PCR analysis of *Gan*-null DRG neurons reveals downregulation of several TFEB targets: TFEB itself, mucolipin-1, LAMP-1, Beclin-1, and cathepsins B and D. Data represent fold change in *Gan*-null DRG neurons of the respective gene compared with WT and normalized to the expression level of GAPDH in 3 independent experiments plotted as mean ± SEM. *P < 0.05, **P < 0.01 by 2-tailed, unpaired Student’s *t* test.

After costaining TFEB and NFL, we found that TFEB was enriched in cytoplasmic NF aggregates when *Gan* was silenced (Figure 9, A and B). Moreover, we found significantly less TFEB in the nucleus of *Gan*-null cells (~33% less than WT). We also found that 14-3-3 proteins coaggregate with NFL in *Gan*-silenced DRG neurons (Figure 9C).

Because less TFEB is in the nucleus of shGan cells from DRG culture, we next tested for its transcriptional activity. To test for this, we performed quantitative RT-PCR, evaluating a few notable targets of TFEB (56); these include TFEB itself (as part of a positive feedback loop), Beclin-1, a scaffold protein crucial for recruitment of autophagy machinery to the autophagosome, and the lysosomal proteins LAMP-1, mucolipin-1, and cathepsins B and D. These TFEB-regulated transcripts were significantly reduced, in line with the functional compromise of TFEB activity (Figure 9D).

## Discussion

NFs accumulate in several neurodegenerative syndromes — Alzheimer disease, Parkinson disease, polyglutamine diseases, and amyotrophic lateral sclerosis, to name just a few (16). Yet, the role of NF aggregation in these diseases has been largely overlooked in favor of disease-specific features. Rare diseases, such as GAN, provide a unique and valuable opportunity to understand the effects of NF aggregation (16). Here, we show that NF accumulations in GAN impair autophagy by disrupting the spatial distribution and transport of autophagic organelles and the master transcriptional regulator TFEB, which is required for lysosome biogenesis and autophagic flux.

The first hint that impaired autophagy contributes to the pathogenesis of human degenerative syndromes came from genetically engineered mice. Mice lacking autophagy-related 5 or 7 (ATG5 or ATG7) in their nervous system, for example, demonstrate progressive neurological deficits and protein aggregation. Patients have also been described with mutations in genes directly linked with autophagic processes (58, 59). These diseases are rare, but there has been a growing appreciation for the role of autophagy in the common neurodegenerative proteopathies such as Parkinson disease. In GAN, autophagy’s role is more complex; clearance of disease-specific proteins that resist ubiquitin-proteasome degradation is attempted via autophagy, while autophagy itself is compromised by a range of cellular events triggered by the misfolded proteins themselves in pathways yet to be completely elucidated (58, 60, 61). In the normal physiological state, NFs undergo autophagic clearance, and the autophagic vesicles surrounding NF aggregates in GAN suggest that autophagy might even be recruited as a salvage pathway to clear NFs (62). Because rapamycin improves autophagic flux in DRG neurons in vitro, we attempted to treat *Gan*-null mice with rapamycin to determine whether the NF accumulation phenotype in mice could be rescued. Unfortunately, these mice suffered rapamycin toxicity (loss of weight and hair; data not shown). It is nonetheless conceivable that other autophagic stimulators might produce fewer side effects and still prove beneficial in treating this disease.

In many of the common proteopathies, disease proteins such as α-synuclein, mutant Huntingtin, and TDP43 are surrounded by NF caps in structures called aggresomes. It is tempting to speculate that the NF caps contribute to the autophagic impairments in these disorders as well. Gigaxonin has recently been shown to degrade ATG16L, a protein involved in autophagosome maturation, and thus might play an independent role as an autophagy regulator (36). In DRG neurons, we did not observe accumulation of ATG16L, but we cannot exclude that such a mechanism might compound the NF-induced autophagic deficits that we observe.

The mechanisms by which NFs interfere with autophagy — changing the localization of autophagic vesicles and binding TFEB through its interaction with 14-3-3 proteins (with downstream ramifications on TFEB’s transcriptional activity) — are in essence distortions of the normal role of NFs to serve as a docking platform. When aggregated, NFs have very distinct biophysical properties from well-distributed WT polymers; they are tightly packed, display a lack of dynamic behavior, and even appear to undergo solid-to-liquid phase transitions (13, 63). Phosphorylation could further affect these properties (13). The mislocalization of autophagic organelles is reminiscent of what has been previously observed with mitochondria in GAN (9). It will now be important to determine the full complement of proteins and organelles affected by NF aggregates in the disease state. Abnormal interactions are likely to be further compounded by inter-organellar co-dependence, exacerbating the pathology. For instance, mitochondrial deficits could limit cellular energy supplies, impairing lysosomal acidification, while abnormal autophagy could affect mitochondrial quality control through mitophagy. Mitochondrial and lysosomal contact sites could serve as additional points of crosstalk between these 2 dynamic organelles (64). Future studies will be required to tease out the complex interactions between signaling pathways, organelle dysfunction, and NF aggregation to determine strategies to best treat GAN and other NF proteopathies.

## Methods

### Sex as a biological variable.

*Gan*-null mice of both sexes show similar behavioral and pathological phenotypes. Therefore, mice of both sexes were used for these experiments.

### Mice.

The generation of *Gan*-null mice has been previously described (18). The animals were housed in a specific pathogen–free facility at Northwestern University.

### DRG cultures.

DRG neurons were isolated from mice using a published protocol, with few modifications (9). Briefly, mouse DRG were dissected from their paraspinal location; they were then placed in cold dissection medium in a microcentrifuge tube (97.5% HBSS Ca^2+^ and Mg^2+^ free, 1× sodium pyruvate, 0.1% glucose, 10 mM HEPES), pelleted by a 10-second centrifugation pulse on a table-top centrifuge, and then washed in the same dissection media and finally harvested by pelleting. Washed ganglia were dissociated at 37°C for 10 minutes, first in 1 mL of Neurobasal media (Gibco) containing 35 U papain/mL followed by a centrifugation pulse and then in 1 mL of Hibernate medium (Gibco) containing 4 mg/mL collagenase type II (Worthington Biochemical) and 4.6 mg/mL Dispase II (Sigma-Aldrich). This was followed by another pulsed centrifugation and wash. The resuspended cells were triturated by pipetting through a P1000 tip in plating medium (Neurobasal media containing penicillin 100 U/mL, 100 μg/mL streptomycin, and 1× GlutaMax [Gibco]). The dissociated cells were separated from any clumps by filtering them through a 100-μm cell strainer (Corning). They were then harvested for plating by a low-speed centrifugation for 5 minutes at 230*g*.

The cells were then cultured on plating medium in 35-mm microwells with a 14-mm glass bottom (MatTek). Each mouse provided sufficient DRG quantities to plate 5 dishes at approximately 80,000 cells per dish. After allowing the plated cells to settle, the plating media were gently aspirated and replaced with 2 mL of prewarmed maintenance medium (plating media supplemented with 1% nerve growth factor). Primary neurons were maintained at 37°C in a humidified 5% CO_2_ atmosphere with addition of 0.5 mL of fresh maintenance media every 3 days. On day 3 in vitro, DRG cultures were transduced with lentivirus encoding shRNA against *Gan* (shGan) or a scrambled control (shScr) using 10 μL of concentrated lentivirus per microwell, prepared as described below.

### Lentiviral constructs.

EGFP derived from pEGFP-C1 (Clontech) was cloned in-frame into the N-terminal domain of the mouse NFL gene (derived from pmNFL; Addgene, 83127) using the NEBuilder HiFi DNA Assembly cloning kit (New England Biolabs, E5520S). The fusion construct was then amplified by PCR and inserted into the multiple-cloning site of the lentiviral vector pLEX using the same NEBuilder cloning kit to generate pLEX-mNFL-GFP. Constructs were validated with Sanger sequencing using primers to sequence over insertion sites.

Autophagic flux was measured by an mRFP-GFP-LC3 construct as described previously (65). Lentiviruses expressing shGan and shScr have been described previously and were obtained from MISSION shRNA systems: shGan (MISSION vector TRCN0000251146, Sigma-Aldrich) and shScr (SHC002, Sigma-Aldrich).

### Lentivirus production and transduction.

HEK293T cells were used to produce all the lentiviruses. HEK293T cells were plated in T75 flasks at a density of 60%–80% in a culture medium of high-glucose DMEM (Gibco, 11965092) supplemented with 10% fetal bovine serum (Gibco, 16140089) and 100 U/mL penicillin/100 μg/mL streptomycin (Gibco, 15140122). The lentiviral construct of interest was cotransfected with the packaging constructs pCMV-VSV-G and pCMV Gag/Pol at a ratio of 10 μg/4 μg/6 μg, respectively, using Lipofectamine 2000 (60 μL; Invitrogen, 11668027). After 4 hours, the growth media of the transfected HEK293T cells were replaced with 10 mL of fresh complete DMEM. Media were collected 48 hours after transfection and clarified by syringe filtration through a 0.45-μm polyvinylidene difluoride membrane (Millex-HV) before concentrating the virus with a Lenti-X concentrator (Clontech, 631231). The concentrated virus was resuspended in PBS and aliquoted into single-use tubes stored at –80°C. The concentrated lentivirus in a volume of 10 μL was delivered to DRG neurons cultured on glass-bottomed microwells. Each new lot of lentiviruses was first tested for at least 50% transduction efficacy in WT DRG cultures before experimental use (as measured by fluorescence of tagged constructs or knockdown of shRNA constructs as determined by qPCR).

### SILAC sample preparation and data analysis.

DRG neurons from P3 WT mice were isolated and plated as previously described (9). In brief, the dissociated neurons were plated on poly-D-lysine coverslips and maintained in Neurobasal media for 13 days. Gigaxonin was silenced using lentivirus expressing shRNA against *Gan* (shGan) or control (scrambled sequence; shScr) after 3 days in culture. In total, 8 dishes of DRG neurons were cultured for SILAC analysis where a standard label-swapping approach was carried out. Here, 2 cultures treated with shScr and 2 cultures treated with shGan were incubated with media containing heavy isotope–enriched arginine and lysine (^13^C^15^N). In parallel, 2 cultures treated with shScr and 2 cultures treated with shGan were incubated with normal culture media. After 13 days in vitro, cells were lysed with 1% SDS–containing 100 mM Tris-Cl and boiled for 10 minutes. Protein concentrations were determined via a bicinchoninic assay (BCA, Pierce). Equal amounts of protein (50 μg) from pairs of heavy and light cultures were mixed at a 1:1 ratio. An in-solution trypsin digestion of proteins was carried out after reduction with 5 mM dithiothreitol at 55°C for 15 minutes and alkylation with 15 mM iodoacetamide for 20 minutes at room temperature. The resulting peptides were fractionated via HyperSep Strong Cation Exchange using 25 mM, 50 mM, 500 mM, 1 M, 2 M, and 4 M KCl, and each fraction was subsequently desalted by C18 ZipTip and dried in vacuo. Each of the samples was resuspended in 0.1% formic acid and analyzed via nano-electrospray ionization on a Thermo Orbitrap Fusion mass spectrometer, where auto MS/MS data were acquired in positive ion mode. Here, solvent A was 0.1% formic acid and solvent B was acetonitrile with 0.1% formic acid. Peptides were resolved using a 60-minute linear gradient where a single MS analysis was done for each fraction. Data analysis was performed using the Thermo Proteome Discoverer software. “Light” samples were Lys0 and Arg0 and “heavy” samples were Lys8 and Arg10. The database search included fixed modifications, such as carbaminomidomethyl (C), and variable modifications such as oxidation (M) and deamination (N, Q). Precursor quantification of pairwise ratios (matched median peptide abundance) and *t* test analysis using no missing channels were used to calculate relative quantification ratios. Significance of shGan/shScr ratios was determined at a threshold of *P* equal to 0.05 or less with Benjamini-Hochberg correction applied to reduce the FDR. Pathway and biofunction analysis of altered proteins were performed using IPA.

### Immunocytochemistry.

DRG neurons on glass-bottomed microwells were fixed in –20°C methanol for 7 minutes, a method which is ideal for fixation of NFs (66). After fixation, the cells were incubated for 1 hour with blocking solution (5% normal goat serum in PBS) and then incubated overnight at 4°C with the relevant primary antibodies diluted in blocking solution. The microwells were then washed twice with PBS containing 0.05% Tween 20 followed by a similar wash twice with PBS (each wash 5 minutes). The cells were then incubated for 1 hour at room temperature with Alexa Fluor–conjugated secondary antibodies and DAPI (1:500 dilution) diluted in blocking solution and washed again as described above. A drop of mounting media (Prolong Diamond; Life Technologies) was added to the microwell and a coverslip was placed. The following primary antibodies were used: neurofilament NFL chicken polyclonal (CPCA-NF-L, Encor), LC3A/B monoclonal (12741, Cell Signaling Technology), LAMP-1 rabbit polyclonal (ab24170, Abcam), GAPDH rabbit monoclonal (2118, Cell Signaling Technology), TFEB rabbit polyclonal (SAB2108453, Labome), TFEB rabbit polyclonal (A303-773A, Bethyl Laboratories), 14-3-3 rabbit polyclonal (51-0700, Cell Signaling Technology), cathepsin B mouse monoclonal (ab58802, Abcam), cathepsin D mouse monoclonal (ab75852, Abcam), and mucolipin-1 rabbit polyclonal (PA1-46474, Invitrogen).

### Live imaging of lysosomes and autophagic flux.

The conditioned media of DRG cultures that would be subjected to lysosomal staining were first removed and kept aside. The cells were then treated with 50 mM LysoTracker (Red DND-99; L7528, Invitrogen) either alone or in combination with 1 μM LysoSensor (Green DND-189; L7535, Invitrogen) in plating media for 30 minutes, after which the media were replaced with the previously stored media (to avoid LysoTracker cytotoxicity). When we wished to visualize NFs, we also transduced the cells 3 days before treatment with pLEX-mNFL-GFP. To measure autophagic flux in living cells, we transduced DRG cultures with mRFP-GFP-LC3 lentivirus 3 days prior to imaging. To measure basal autophagic flux, DRG cells were treated either with vehicle or 400 mM bafilomycin A1 (Sigma-Aldrich) for 24 hours, after which Western blots were run to monitor LC3-II levels. To measure flux in the presence of rapamycin, the DRG cells were first treated with 100 nM rapamycin (Sigma-Aldrich) for 20 hours and then bafilomycin A1 (400 nM) was added for an additional 4 hours before lysis. Autophagic flux was measured as described previously (52), with the autophagic flux in WT neurons normalized to 1.

### Light microscopy.

Resonant scanning confocal microscopy was performed using a Nikon A1R+ platform equipped with a 100× oil-immersion objective and PerfectFocus focal drift compensation mechanism with automated XY stage. The green fluorophores were excited using laser lines set at 488 nm and emission filters set at 525–550 nm; the red fluorophores were excited using a laser line set at 561 nm with emission filters set at 600–650 nm. The confocal pinhole size was fixed at 1.2 times the size of the Airy disc of the red channel. For live cell microscopy, images were captured at a single XYZ position every second for 3 minutes using 8 frame averages to improve the signal-to-noise ratio. For fixed cell microscopy, images were acquired in Galvano mode. Image resolution was optimized using the Nyquist criterion by the Nikon Elements software.

### Transmission electronic microscopy.

DRG cultures were fixed in 0.1 M sodium cacodylate buffer (pH 7.3) containing 2% paraformaldehyde and 2.5% glutaraldehyde. They were then processed and embedded in resin blocks which were then sectioned, stained, and imaged as previously described (9).

### Western blotting.

The DRG media of each microwell culture were aspirated and replaced with 70 μL RIPA buffer containing 1% protease inhibitor. The cells were gently scraped with the pipette tip and the lysates were collected in microcentrifuge tubes. To ensure adequate lysates for Western blotting, typically 5 dishes of each experimental sample were pooled. Protein concentrations were determined using the Pierce BCA assay (Thermo Fisher Scientific). Protein extracts were prepared with Laemmli buffer, warmed at 95°C for 5 minutes, and separated in gradient SDS-polyacrylamide gels by electrophoresis. The proteins were then electrophoretically transferred to nitrocellulose membranes. Membranes were blocked at room temperature for 1 hour using 5% blocking solution and blotted sequentially with the appropriate primary antibody (overnight 4°C) and the horseradish peroxidase–conjugated secondary antibodies (2 hours at room temperature). Between primary and secondary antibody incubation, membranes were washed 3 times, 10 minutes each, with PBS with 0.1% Tween 20. Similarly, membranes were washed after secondary antibody incubation before protein detection using the SuperSignal West Pico Chemiluminescence Detection kit (Thermo Fisher Scientific). Images were acquired with a Bio-Rad Chemidoc XRS+ gel imaging system and analyzed with ImageJ/Fiji software (NIH). Relative protein quantification was carried out by measuring the area under the curve (AUC) normalized to values of loading controls (GAPDH or β-tubulin) as indicated in the figure legends.

### Quantitative RT-PCR.

We isolated RNA from DRG neurons cultured from 2-month-old mice. DRG cultures were washed with 500 μL PBS and the cells were harvested in 70 μL 0.05% trypsin-EDTA solution (Gibco, 25300-054), pelleted by centrifugation at 500*g* for 3 minutes, and resuspended in 75 μL QIAzol (Qiagen, 79306). Cell suspensions were then lysed by sonication and RNA was isolated (RNAeasy Plus Universal Minikit, Qiagen). We generated cDNA from the isolated RNA using iScript Reverse Transcription Supermix (Bio-Rad), after which we performed quantitative PCR reactions using a Bio-Rad CFX-96. Relative fold-change was determined as ΔΔCt of mean values using the GAPDH housekeeping transcript and comparing experimental ΔCt values to those of control. The sequences of the primers used for qPCR are as follows: GAPDH F: AACAGCAACTCCCACTCTTC, GAPDH R: CCTGTTGCTGTAGCCGTATT; TFEB F: CAGAAGCGAGAGCTAACAGATG, TFEB R: GAACCTGCGTCTTCTCTCAATTA; Beclin-1 F: GAGGAATGCACAGACACTCTT, Beclin-1 R: CATCTGCTCTAGGATCTCCAAAC; cathepsin B F: GTTCCCTCCTACACTTCCTTTC, cathepsin B R: TGTTCAGGGTCCTCTTCTCT; cathepsin D F: CTACCTGAACGTCACTCGAAAG, cathepsin D R: GATGTCCCTGTGTCCACAATAG; LAMP-1 F: GACCCTGAAAGTGGAGAACAA, LAMP-1 R: GGGCATCAGGAAGAGTCATATT; mucolipin-1 F: CAGTGAGGACTTGGACTTCTTG, mucolipin-1 R: GATGAGGCTCTGCAGGTTAAT.

### Image analysis.

All images were quantified with ImageJ/Fiji software. For particle analysis (defining lysosomal compartments and measuring within specific regions of interest [ROIs]), images were prepared by applying general background subtraction on independent channels before applying a trous wavelet transformation (Ihor Smal, A Trous Wavelet Filter) on 3 scales. The output was then converted to a binary mask and Fiji’s built-in particle analysis feature was used to define ROIs. These ROIs served as measurements of lysosome number and size within a cell and were used as bounds for measurements of LysoSensor intensity within LysoTracker-defined compartments (intensity was determined using the multi-measure plugin).

### Statistics.

All values represent the mean ± SEM of the number of replicates indicated in the figure legends. All analyses were performed using GraphPad Prism software. Student’s *t* test was used to compare 2 groups, ANOVA was used to compare more than 2 groups, and statistical significance was defined as *P* less than 0.05. See figure legends for more details.

### Study approval.

Experiments were performed in accordance with the NIH *Guide for the Care and Use of Laboratory Animals* (National Academies Press, 2011), with protocols approved by Northwestern University’s Institutional Animal Care and Use Committee.

### Data availability.

The list of proteins identified in MS analysis from database searches is available in Supplemental Tables 1 and 2. Raw data underlying figures can be found in the supplemental Supporting Data Values file. Any other data will be made available with request in accordance with Northwestern University’s data sharing policy. All MS data have been deposited to the MassIVE data repository with the dataset identifier MSV000091762.

## Author contributions

JMP assisted by JZ and MV performed most of the cell biological experiments. JMP wrote the first draft of the manuscript. EI, MRP, and JNS performed the proteomic experiments and data analysis. SP and CP performed autophagy flux experiments. CP and NS helped in the conceptual analysis and writing of the manuscript. PO supervised the entire work, analyzed and interpreted the data, and wrote the manuscript with input from all the authors.

## Supplementary Material

Supplemental data

Unedited blot and gel images

Supporting data values

## Version 1 03/10/2025

Electronic publication
