# Supplementary material for: Neurofilament accumulation disrupts autophagy in giant axonal neuropathy
Source: JCI Insight. 2025 Mar 10;10(5):e177999. doi: 10.1172/jci.insight.177999 (PMC11949051; doi:10.1172/jci.insight.177999)

p62 and LC3 – Raw data for Fig 7D

Blot for p62

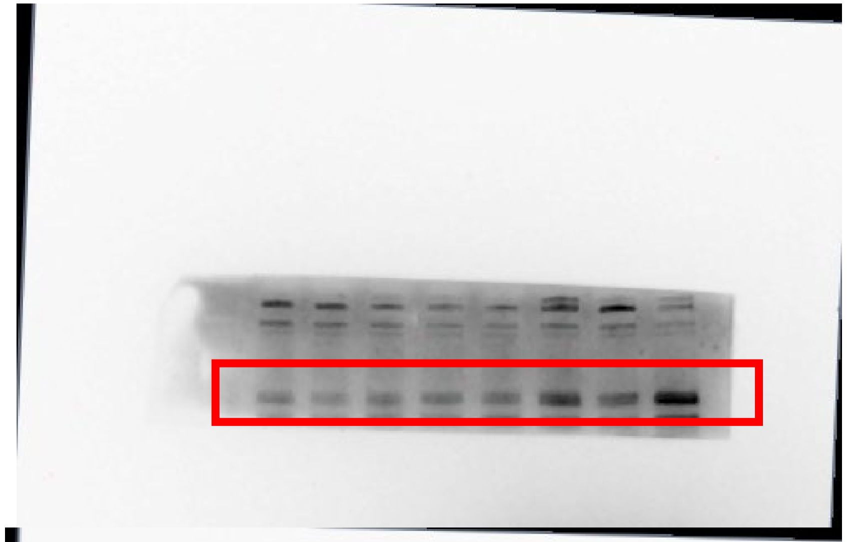

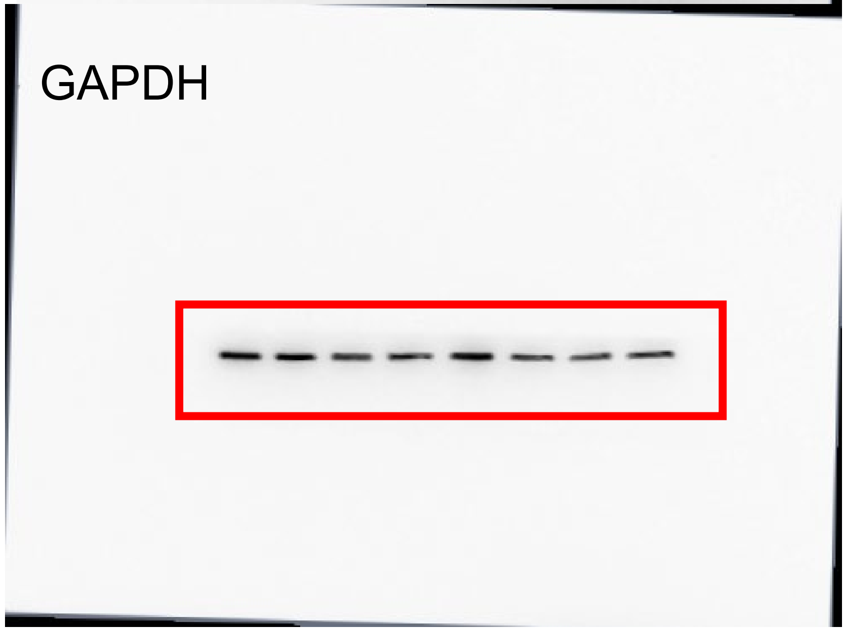

Blot for LC3

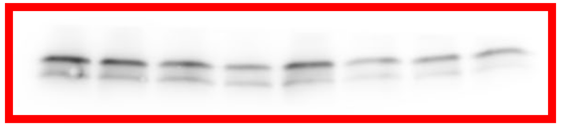

GAPDH

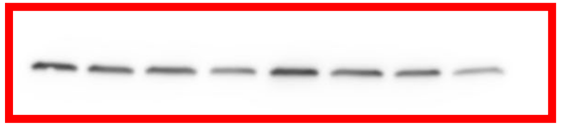

Autophagic Flux – Raw data for Fig 7E

Set 1

LC3

Less exposure

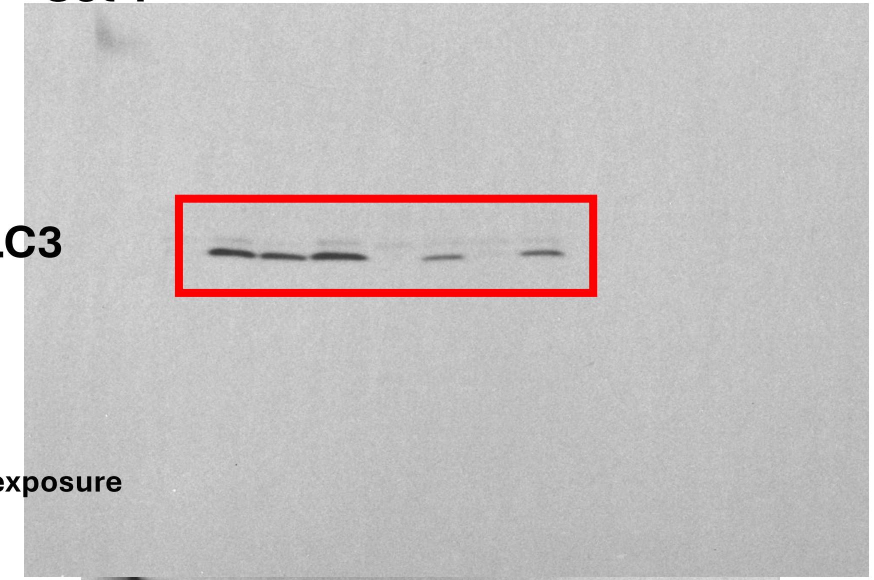

LC3

More exposure

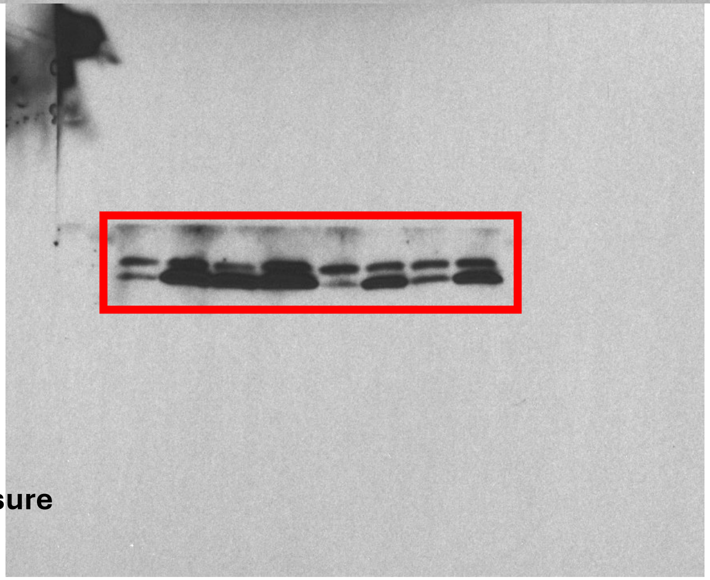

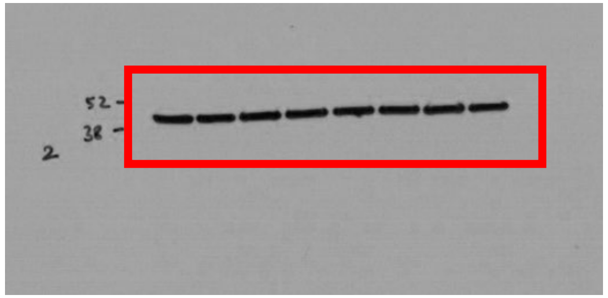

GAPDH

Autophagic Flux – Additional experiments

Set 2

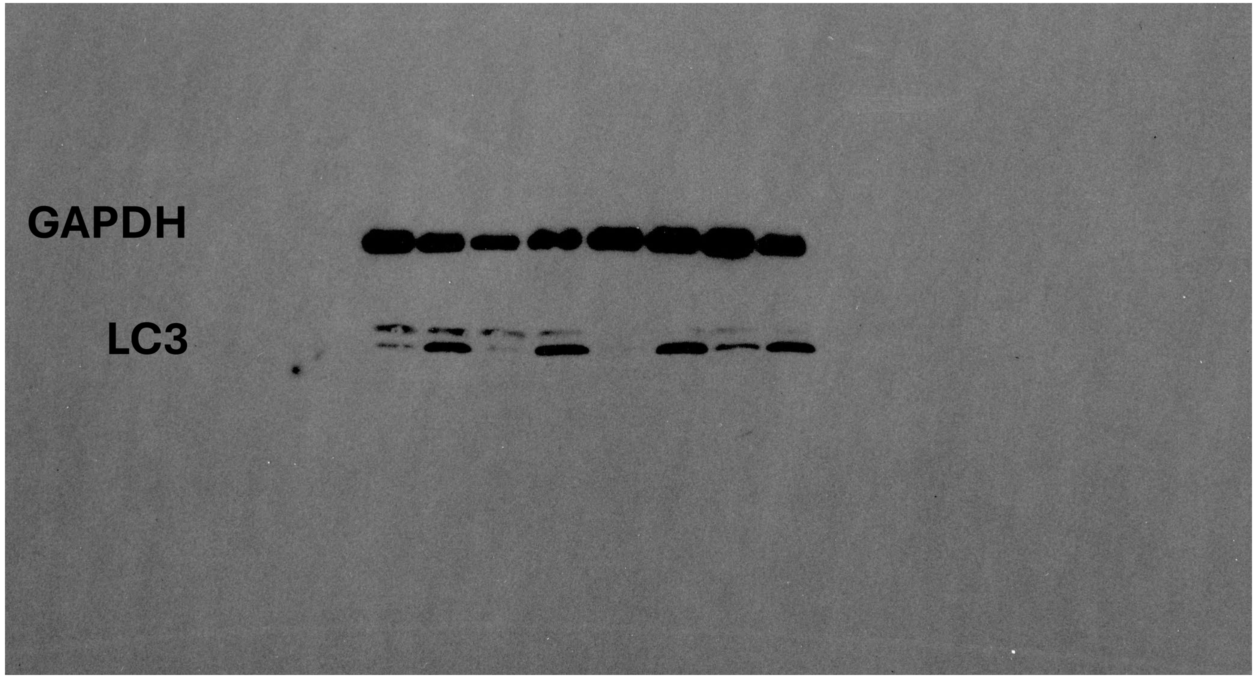

Set 3

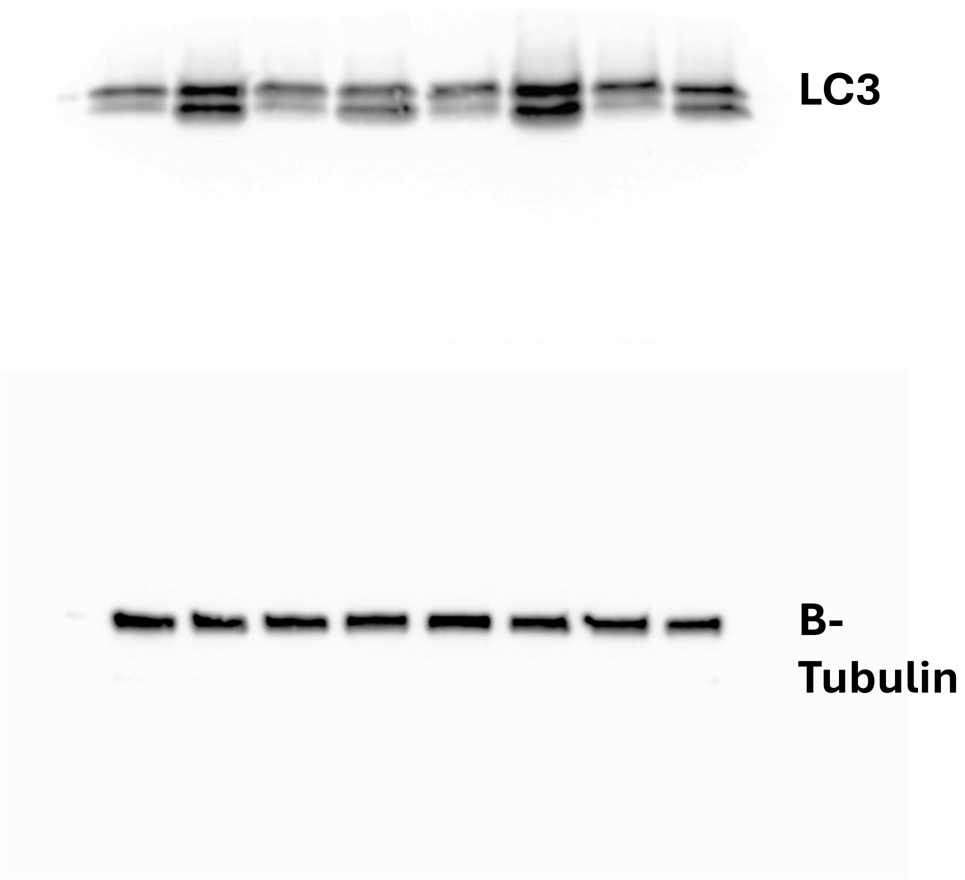

Supplement: Unedited blot and gel images [file jciinsight-10-177999-s054.pdf]
